# Inadvertent complication of the Senning procedure

**DOI:** 10.1007/s12471-016-0840-8

**Published:** 2016-05-23

**Authors:** S. Moustafa, N. Merchant, M. S. Connelly, D. J. Patton

**Affiliations:** Division of Cardiovascular Diseases, Mayo Clinic Arizona, Scottsdale, AZ USA; Department of Radiology, University of Calgary, Calgary, AB Canada; Adult Congenital Heart Disease Clinic, University of Calgary, Calgary, AB Canada; Section of Pediatric Cardiology, Alberta Children’s Hospital, University of Calgary, Calgary, AB Canada

A 34-year-old man with complete transposition of the great arteries (D-TGA) presented for the first time to our clinic. He previously underwent balloon atrial septostomy (Rashkind procedure) early after birth followed by a Senning procedure at the age of 2 months together with ligation of a patent ductus arteriosus. He has been asymptomatic since his surgery.

Transthoracic echocardiogram was technically challenging yet revealed a mildly dilated, hypertrophied systemic right ventricle with mild systolic dysfunction, normal sub-pulmonary left ventricular size and systolic function with no baffle leak or stenosis. No significant valvulopathy was noted (Movie). Cardiovascular magnetic resonance (CMR) and cardiac computed tomography (CCT) showed a moderately hypertrophied systemic right ventricle with normal systolic function (right ventricular ejection fraction 48 %). The sub-pulmonic left ventricle was dilated with normal systolic function. Systemic venous baffles were patent. The pulmonary venous portion of the baffle was patent. The main pulmonary artery and both branches were dilated (Fig. 1).

**Fig. 1:**
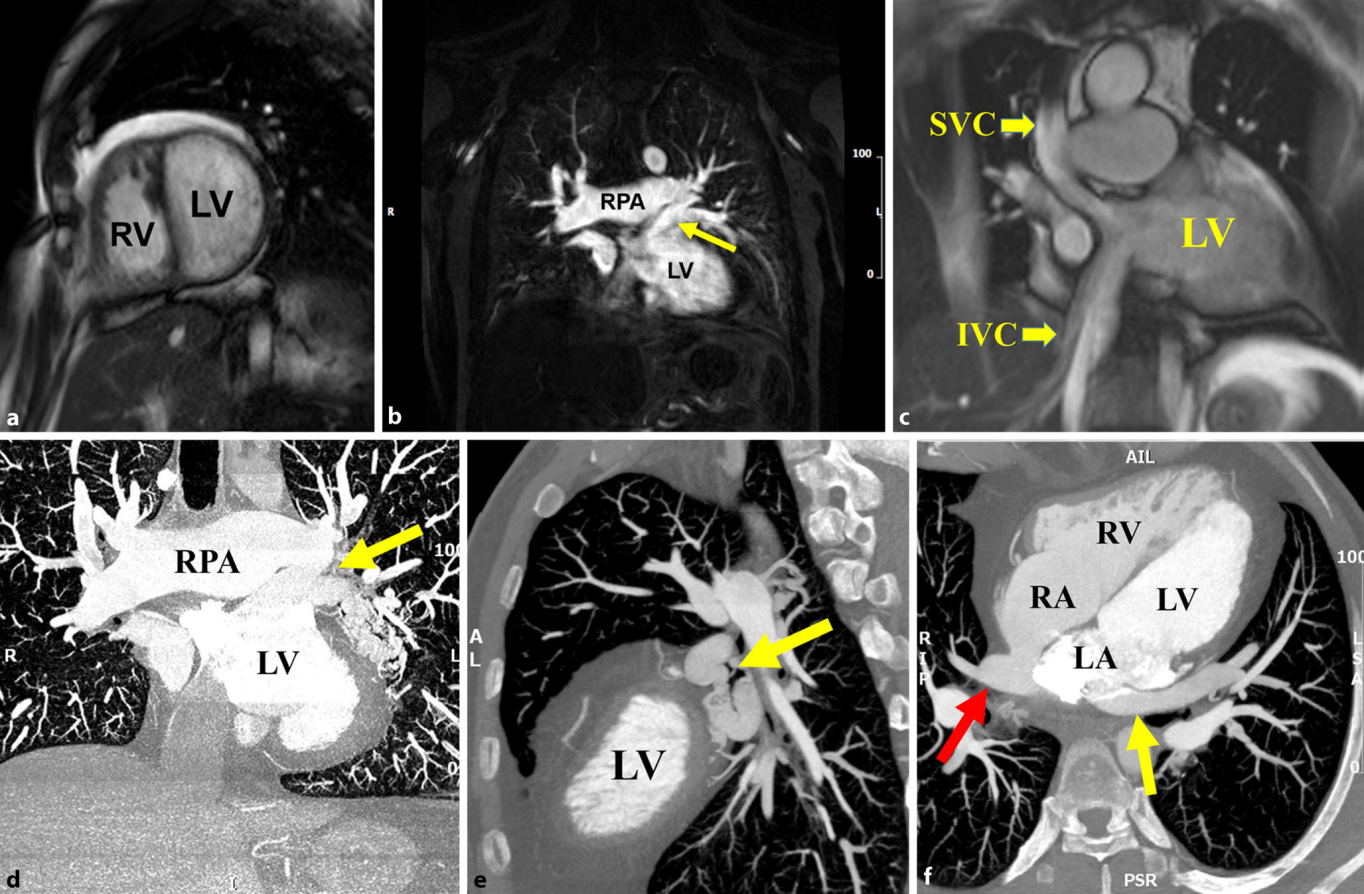
**a** CMR short-axis steady-state free precession (SSFP) coronal image showing hypertrabeculated systemic RV with dilated LV. **b** CMR angiography revealing the left lower PV draining superiorly to a larger common left-side PV which in turn connects to the superior aspect of the left atrium (*arrow*). Right pulmonary artery (RPA) is dilated. **c** CMR SSFP coronal image revealing patent superior (SVC) and inferior vena cava (IVC) limbs of the Senning baffle. **d** CCT maximal intensity projection coronal image showing a tortuous varix of the left lower PV draining superiorly to a larger common left-side PV which in turn connects to the superior aspect of the left atrium. RPA is dilated. **e** CCT sagittal view revealing the tortuous varix (*arrow*). **f** CCT axial view showing the site of anastomosis between the left atrium and anomalous PV connection (*yellow arrow*). Patent pulmonary venous baffle was noted (*red arrow*)

CMR angiography and CCT uncovered a tortuous varix of the left lower pulmonary vein (PV) draining superiorly to a larger common left-side PV which in turn connected to the superior aspect of the left atrium to the left of the Senning baffle (residual left-to-right shunt). Qp:Qs ratio was estimated at 1.9 by flow data and 2.1 by stroke volume analysis. The right PVs were dilated (Fig. 1).

Due to the presence of significant residual left-to-right shunt with a dilated sub-pulmonic ventricle, cardiac catheterisation and potential corrective surgery were recommended. However, the patient declined the procedure.

To the best of our knowledge, this is the first case in the literature exploring an unusual anomalous PV connection in a patient with D‑TGA following atrial switch procedure. We speculate that the patient had this anomalous connection since birth but may have been overlooked during surgical correction [1–3].

## Caption Electronic Supplementary Material

Transthoracic echocardiogram apical 4‑chamber view showing mildly dilated hypertrophied RV with mild systolic dysfunction and normal LV systolic function.

## References

[1] Talwar S, Rajashekar P, Reddy VA (2013). Transposition of great arteries and partial anomalous pulmonary venous drainage. Asian Cardiovasc Thorac Ann.

[2] Seliem MA, Bouholaigah IH, Palileo MR (2004). Complete transposition of the great arteries and total anomalous pulmonary venous connection with a small atrial septal defect: a rare combination resulting in balanced pulmonary systemic circulations. Ann Saudi Med.

[3] Ueda Y, Miki S, Okita Y (1994). Transposition of the great arteries associated with total anomalous pulmonary venous return. Ann Thorac Surg.

